# Hydrocephalus in Guillain barre syndrome: A case report and review of the literature

**DOI:** 10.1097/MD.0000000000018638

**Published:** 2020-04-17

**Authors:** Ammar Taha Abdullah Abdulaziz, Dong Zhou, Jin Mei Li

**Affiliations:** a Neurology Department, West China Hospital, Sichuan University; b Professor, Head of Neurology Department, West China Hospital, Sichuan University; c Professor, Neurology Department, West China Hospital, Sichuan University, PR China.

**Keywords:** dysautonomia, Guillian-Barré syndrome, hydrocephalus, respiratory failure

## Abstract

**Rationale:**

Guillian-Barré syndrome (GBS) is a devastating autoimmune disorder characterized by progressive ascending weakness, areflexia with or without autonomic and sensory disturbances. Hydrocephalus is a rare but well-documented complication in patients with GBS. However, due to the rarity of this condition, no treatment guideline for GBS with hydrocephalus is currently available.

**Patient concerns:**

We describe a 23-year old woman with a history of bilateral limbs pain followed by dysarthria, dysphagia, severe quadriplegia 0/5, areflexia, loss of consciousness and dysautonomia. Neuroimaging studies revealed enlarged lateral ventricles; while Electromyography demonstrated demyelination and nerve injury. Lumbar puncture results showed elevated protein level 2.6 g/L; normal glucose and cell count.

**Diagnosis:**

GBS with hydrocephalus.

**Interventions:**

The patient was started on intravenous immunoglobulin for 5 consecutive days followed by endotracheal intubation and supportive therapy including osmotherapy and CSF drainage.

**Outcomes:**

At 2 months after admission, the patient stopped choking and had a significant improvement in muscles’ strength (grade 4) and pain; then was discharged. On 1 year post-discharge follow-up, CT has revealed a significant improvement of hydrocephalus, and the patient has completely returned to the normal baseline.

**Lessons:**

Respiratory failure is the strongest predictor of concurrent hydrocephalus in patients with GBS. The prognosis of hydrocephalus in patients with GBS is usually good, and it can be medically treated; thereby shunt surgery is rarely required.

## 1 Introduction

Guillian-Barré syndrome (GBS) is a serious immune-mediated neurological disorder characterized by progressive symmetrical ascending weakness in the 4 extremities, areflexia with or without autonomic and sensory disturbances.^[1]^ In the acute stage of GBS, hydrocephalus and increased intracranial pressure are uncommon; but well-recognized complications that present in approximately 4% of the cases.^[2]^ The widely accepted underlying mechanism for hydrocephalus formation is reduced CSF absorption due to a high protein concentration that blocks the arachnoid villi.^[3]^ We report a rare case of GBS complicated with hydrocephalus where accurate diagnosis and early management led to an excellent outcome.

## 2 Case presentation

A 23-year old woman presented with intermittent bilateral foot pain for 2 months, which had dramatically exacerbated over a period of 1 month without numbness, blurred vision or fever. Ten days prior to admission, she developed bilateral hand pain and diffused progressive ascending weakness of all the four limbs, which left her bedrriden. Her history was negative for any recent upper respiratory track or gastrointestinal infection; and she did not have immunization recently.

Upon admission, the patient was alert and oriented; general examination was unremarkable except for high blood pressure 161/128 mmHg; while neurological examination showed decreased muscle strength 4/5 in both extremities with hypo-reflexia. There were no sensory symptoms or any sign of respiratory muscle involvement.

**Figure 1:**
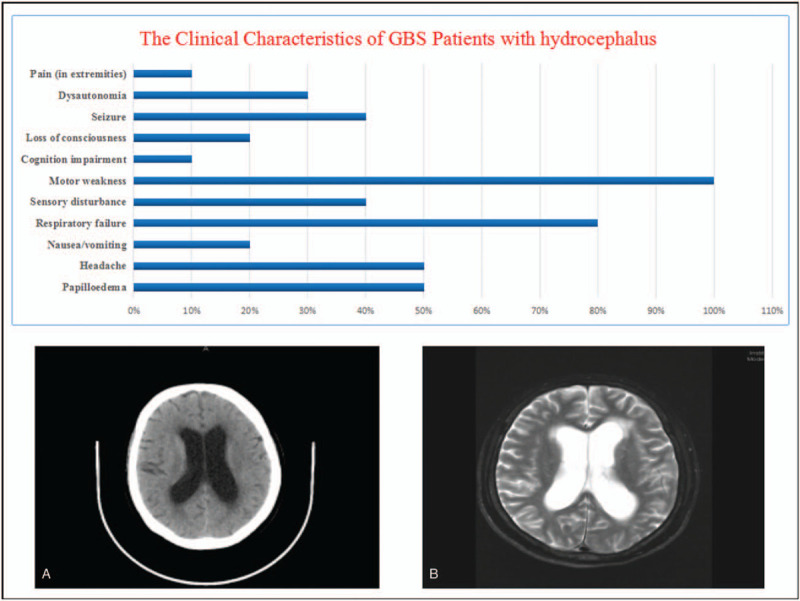
The diagram shows the clinical characteristics of Guillian-Barré syndrome patients with hydrocephalus; while the computed tomography at admission reveals bilateral ventricular enlargement (A); and the MRI at 2 months post-admission reveals persistent enlargement of the lateral ventricles (B).

Few days after admission, her condition deteriorated with choking, dysarthria, dysphagia, severe quadriplegia 0/5, areflexia and episodes of loss of consciousness. Followed by unstable blood pressure, fluctuating heart rate and excessive sweating. Complete blood count, biochemistry panel, thyroid function test, anti-neutrophil cytoplasmic antibody and immunology panel were normal. Head computed tomography (CT) showed enlarged lateral ventricles Figure 1(A); while head CT angiography, chest CT and abdominal CT were unremarkable. Bone marrow biopsy was normal. Electromyography and nerve conduction study demonstrated characteristic findings of demyelination and nerve injury.

Lumbar puncture (LP) was performed, with 140 mmH_2_o opening pressure, elevated protein level 2.6 g/L, normal glucose and cell count. Oligoclonal immunoglobulin bands were not present. Serology was negative for hepatitis B, hepatitis C, cytomegalovirus and HIV. Based on the clinical features, laboratory and electrophysiological findings, a diagnosis of Guillain Barre syndrome was made.

**Figure 2:**
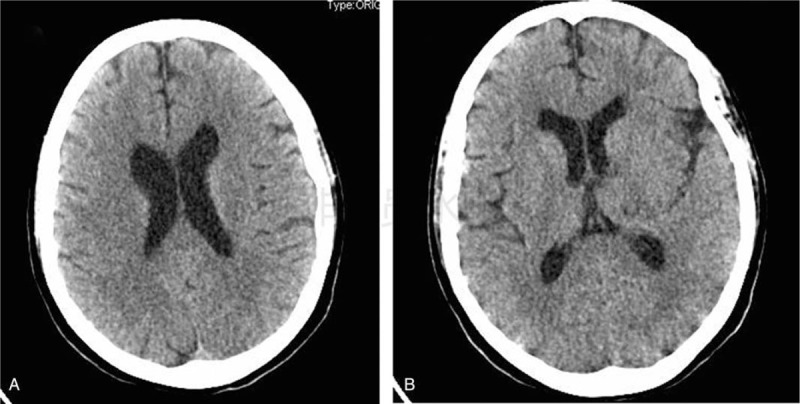
The computed tomography imaging at 1-year post-discharge (A, B) shows near normal brain findings.

On the 12th day of admission, the patient was placed on mechanical ventilation due to severe pneumonia and respiratory failure. The patient started with intravenous immunoglobulin (IVIg) at a dose of 0.4 g/kg daily for 5 days, mannitol 125 ml q6h, antibiotics and supportive therapy. The patient's condition gradually became relatively stable over the following one week, then she had a sudden collapse due to cardiac arrest; after an emergency Cardiopulmonary resuscitation the patient's vital signs returned to the baseline, however, she remained ventilator dependent for 21 days. One month after immunotherapy (Over 40 days post-admission), her condition stabilized obviously, thereby the ventilator was removed. One week later, the patient started to experience mild improvement (reduced pain and weakness). At 45 days post-admission, a follow-up LP was performed, which revealed a very high level IgG protein synthesis 22.338 mg/day. An external lumbar drainage was placed for 2 weeks; with the patient's symptoms continued to gradually improve over time. At 2 months post-admission, the patient was able to drink and eat without choking; limbs’ pain was reduced significantly along with an obvious improvement in muscles’ strength (grade 4); a follow-up magnetic resonance imaging revealed a persistent enlargement of the lateral ventricles Figure 1(B); the patient was discharged with nerve supporting therapy as well as physiotherapy. At 4 months post-discharge, she was able to walk without assistance. On the last Follow-up (1 year post-discharge), the patient was almost completely recovered, and a significant improvement of hydrocephalus was noted on her last brain CT, Figure 2.

## 3 Discussion

Hydrocephalus is a rare complication of GBS that can appear at any time during the disease course. To the best of our knowledge this is the tenth case to be reported in the last 60 years where hydrocephalus is associated with GBS, thus making it rare and notable. Janeway and Kelly were the first group to report the association of hydrocephalus with GBS.^[4]^ As then a number of case reports summarized by Liu et al has been available.^[5]^ All the published cases in literature as well as this case are summarized in Table 1. The average age of GBS patients with hydrocephalus was 19 with a range from 1 month to 67 years; Male to female ratio was 2:1. The common clinical presentations in these patients were headache in 5 out of 10 patients, motor weakness in all patients, papilloedema in 5/10, respiratory insufficiency in 8/10, dysautonomia in 3/10, sensory disturbance in 4/10 and other symptoms^[2,4–11]^; Figure 1.

**Table 1:**
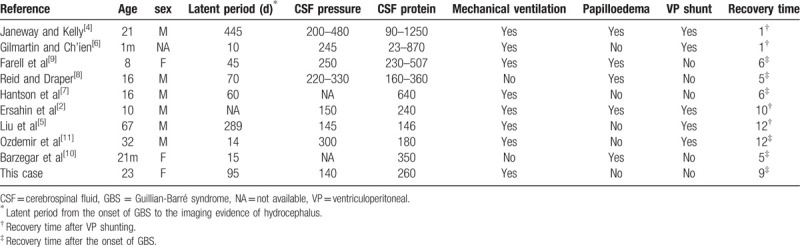
The reported cases of GBS with hydrocephalus in literature.

Hydrocephalus is a seldom occurrence that can be missed in patient with GBS who develops respiratory failure and becomes ventilator dependent, due to their loss of verbal communication. Therefore, many researchers recommended brain imaging studies in GBS patients with respiratory failure, papilledema, seizure, extremely high cerebrospinal fluid (CSF) protein >300 mg/dL, high CSF pressure >200 mm H_2_O, headache, nausea, vomiting, or cognitive deficits.^[5,12]^

The pathophysiologic mechanism of increased intracranial pressure and hydrocephalus in such cases remains uncertain; with three hypothesized theories:

(1) increased CSF protein concentration and osmotic pressure;
(2) obstructed CSF absorption; and
(3) brain edema.^[3,6,13]^

However, most authors proposed the idea of reduced CSF absorption as the main cause of intracranial hypertension with subsequent hydrocephalus, which can improve spontaneously,^[7–9]^ or be relieved by the diversion of CSF.^[2,4,6]^

Beside the usual symptoms, our patient had experienced pain in all extremities. Although, Pain is an important clinical feature of GBS, it was not emphasized in consensus diagnostic criteria. One of the largest prospective studies of pain in GBS reported that pain can occur at anytime throughout the course of illness. In the prodromal phase, pain develops in 36% of patients, occurring most frequently in the extremities and back. They also found that the prevalence of pain is maximal during the first 4 weeks of the disease, and then gradually declines in both prevalence and severity over time.^[14]^

In almost all the previously reported cases, hydrocephalus occurred after GBS onset except for 1 case reported by Ozdemir et al. In which acute hydrocephalus preceded GBS symptoms.^[11]^ Therefore, GBS should be considered in patients with acute hydrocephalus, especially when complicated with paralysis.^[11]^ The latent period from disease onset until the neuroimaging confirmation of hydrocephalus is ranged from 10 to 445 days.^[14]^ Our patient had the first head CT scan 3 months after GBS onset Figure 1(A).

Respiratory failure had been reported as the most common complication in GBS patients, affecting 20% to 30% of all cases^[15]^; however, the prevalence rate is extremely higher in GBS patients with subsequent hydrocephalus. We found that respiratory failure was the strongest predictor of underlying hydrocephalus among GBS patients, which is consistent with the previously reported data.^[5]^ Our patient remained ventilator dependent for over 20 days. Papilledema also infrequent finding in GBS, usually associated with elevated CSF protein and symptoms related to increased ICP.

Due to the rarity of this condition and the lack of updated literature on this topic; no available treatment guidelines for GBS with hydrocephalus is exist. Therefore, CSF tap test or the external lumbar drainage procedure is highly recommended to predict the outcome of shunt surgery in hydrocephalus.^[16]^ However, the prognosis of hydrocephalus in GBS is usually good and it could be successfully treated medically; thereby shunt surgery is rarely required, which is consistent with our findings.^[5,9–10]^ It has been found that recovery time in patients who underwent surgical shunt varied from 1 to 12 months and for those who were treated medically this period was 5 to 6 months.^[5]^ Our patient was successfully managed with medications (immunotherapy). At 4 months follow up she was able to walk without assistance.

## 4 Conclusion

Hydrocephalus is a rare but well-recognized complication of GBS; that can appear at any time during the disease course.

Respiratory failure is the strongest predictor of concurrent hydrocephalus in patients with GBS.

The prognosis of hydrocephalus in GBS is usually good, and it can be medically treated; thereby shunt surgery is rarely required.

## Author contributions

**Conceptualization:** Ammar Taha Abdullah Abdulaziz, Jin Mei Li.

**Data curation:** Ammar Taha Abdullah Abdulaziz.

**Resources:** Ammar Taha Abdullah Abdulaziz.

**Supervision:** Jin Mei Li.

**Validation:** Dong Zhou, Jin Mei Li.

**Writing – original draft:** Ammar Taha Abdullah Abdulaziz, Dong Zhou.

**Writing – review & editing:** Dong Zhou.
